# Retained Guidewire Following Perforator Vein Ablation

**DOI:** 10.7759/cureus.117445

**Published:** 2026-10-04

**Authors:** Soumith Sanka, Jesse Chait, Enrico Ascher, Anil Hingorani

**Affiliations:** 1 Internal Medicine, Michigan State University College of Osteopathic Medicine, East Lansing, USA; 2 Vascular Surgery, Mayo Clinic, Rochester, USA; 3 Vascular Surgery, New York University Langone Hospital-Brooklyn, Brooklyn, USA

**Keywords:** chronic venous disease, complications, endovascular, endovenous thermal ablation, perforator vein, retained guidewire

## Abstract

Wire embolization is a rare but potentially serious complication of endovascular procedures. Endovenous thermal ablation is a minimally invasive procedure widely used to treat chronic venous insufficiency. Herein, we report a case of guidewire embolization into the posterior tibial and popliteal veins during endovenous laser ablation treatment that was successfully managed with open surgical retrieval. Because of the extensive length and location of the retained guidewire, inability to obtain reliable distal venous access, and concern for further migration or venous injury with percutaneous manipulation, open surgical retrieval was selected. The guidewire was successfully removed through popliteal vein exploration without perioperative complications. This case highlights the importance of recognizing retained intravascular foreign bodies promptly and maintaining open surgical retrieval as an option when endovascular techniques increase procedural risk.

## Introduction

Wire embolization is a rare but potentially serious complication of endovascular procedures and can result in vascular injury, thrombosis, infection, or severe arrhythmias if not promptly recognized and managed. Retained intravascular foreign bodies, including guidewires, catheter fragments, pacemaker wires, and inferior vena cava (IVC) filters, have been described across a variety of procedures, with retrieval generally recommended when feasible to minimize the risk of subsequent complications [1]. Although the decision to retrieve a retained foreign body should be individualized based on the patient’s symptoms, overall health and life expectancy, location and characteristics of the foreign body, and risks associated with retrieval, conservative management may be reasonable in certain asymptomatic patients with limited life expectancy [2].

Endovenous thermal ablation has become an established minimally invasive treatment for chronic venous insufficiency associated with venous reflux. Perforator veins are relatively small vessels that traverse the deep fascia and connect the superficial and deep venous systems. When perforator veins are incompetent, they may contribute to advanced chronic venous disease and can be treated using several minimally invasive techniques [3]. Endovenous laser ablation is a catheter-based technique where thermal energy is delivered within the target vein to achieve closure. Because perforator veins are relatively small and anatomically variable, catheter and guidewire manipulation may be technically demanding. Moreover, their communication with the deep venous system creates potential for device migration. Depending on the techniques and devices used, guidewire placement and manipulation may be required before positioning the ablation catheter within the target vein. Although endovascular retrieval is generally preferred when an intravascular foreign body is identified, feasibility and safety depend on the location, length, and configuration of the retained object, the patient's vascular anatomy, and the ability to obtain appropriate endovascular access [4].

Here, we present a case of extensive guidewire retention within the posterior tibial and popliteal venous system during endovenous laser ablation of a perforator vein. The guidewire extended across multiple deep venous segments from the distal tibial region through the popliteal region and proximally toward the femoral region. Because distal tibial and popliteal venous access could not be obtained and manipulation of the long intravascular wire was considered to carry a risk of further migration or venous injury, the treating team proceeded with open popliteal vein exploration and successful guidewire removal.

Although retained intravascular foreign bodies are generally approached using endovascular retrieval techniques, there are circumstances in which open surgical retrieval may provide a safer alternative. This case highlights the importance of prompt recognition of retained intravascular foreign bodies and demonstrates that open surgical retrieval remains an important and effective option when endovascular retrieval is unsafe or technically impractical.

## Case presentation

A 44-year-old woman was brought to the emergency department by ambulance after being referred by a private practice general surgeon who had lost control of a guidewire during an attempted endovenous laser ablation of a perforator vein. The initial endovenous procedure was performed at an outside practice prior to transfer to our institution. The exact manufacturer, model, diameter, total length, coating characteristics, and associated sheath or catheter size of the retained guidewire were not documented in the medical records available for review. The patient denied any pain, shortness of breath, subjective fever, nausea, or vomiting. The surgeon advised her to avoid bending the affected knee. Her past medical history was significant for chronic venous insufficiency. 

Upon physical examination, the patient had edema and decreased range of motion of the left lower extremity. She had underlying chronic venous insufficiency with pre-existing lower-extremity edema; peri-procedural tumescence was also present and subsequently limited transcutaneous duplex visualization of the retained wire. The skin appeared pale, likely secondary to the local swelling and venous insufficiency, rather than arterial compromise. Distal pulses were palpable, and motor function of the foot and toes remained intact.

Plain radiographs of the left lower extremity confirmed the presence of a retained guidewire, spanning from the midshaft of the femur to the distal aspect of the tibia (Figures 1A, 1B, 1C).

**Figure 1:**
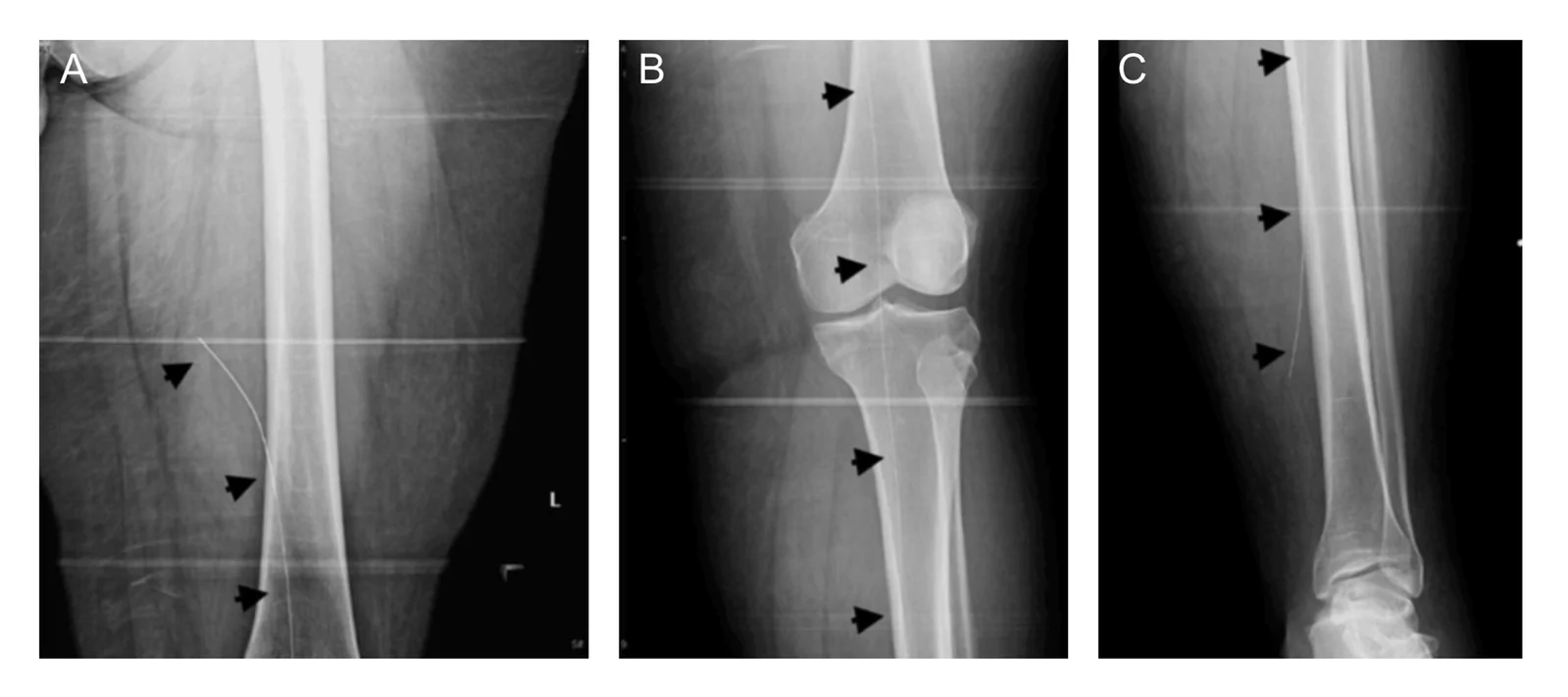
Serial radiographs of the left lower extremity demonstrating the retained guidewire The radiopaque guidewire is identified by arrows and extends from the distal tibial region through the popliteal region to the mid-femoral region. Panels A-C demonstrate the proximal, middle, and distal extent of the retained guidewire, respectively.

After discussion with the patient, the decision was made to proceed with open surgical retrieval. 

Although retrieval through central venous access using a snare or other endovascular retrieval device is an established option for migrated intravascular foreign bodies, no percutaneous retrieval attempt was performed in this case. The treating team favored direct popliteal exposure because the guidewire extended across multiple deep venous segments, distal tibial and popliteal access could not be obtained, and manipulation of the long wire without distal control was considered to carry a risk of further migration or venous injury. This represented a case-specific operative decision rather than a general contraindication to femoral or internal jugular endovascular retrieval.

Intraoperative transcutaneous duplex ultrasonography (DUS) was initially unable to clearly visualize the wire due to excessive edema and tumescence in the leg; therefore, fluoroscopy was utilized to localize the wire. A longitudinal incision was placed on the medial aspect of the distal thigh, and electrocautery was used to dissect the subcutaneous tissue to expose the structures overlying the popliteal artery and vein. At this point, DUS confirmed that the wire had remained within the venous system. The procedure was performed under general anesthesia. Estimated blood loss was approximately 50 mL, and total operative time was approximately 145 minutes (2 hours 25 minutes).

The patient was systemically anticoagulated with intravenous heparin, and proximal and distal control of the popliteal vein was obtained using DeBakey clamps. A transverse venotomy was performed, and the retained guidewire was removed. Vessel loops were placed, and the venotomy was closed using a 6-0 polypropylene suture. After hemostasis was achieved, the wound was irrigated and closed in layers. A sterile dressing was applied, and the patient was extubated and brought to the recovery room in stable condition. 

Postoperatively, the patient was started on a continuous intravenous heparin infusion at 500 units/hour. The available records do not document continuation of therapeutic anticoagulation or prescription of an oral anticoagulant at discharge.

The patient was discharged within 24 hours following the procedure. At her five-and-a-half-year follow-up, the patient did not experience any procedure-related complications or other untoward effects. She continued to have lower extremity edema secondary to chronic venous insufficiency. 

## Discussion

Obtaining intravascular access via the Seldinger technique [5] is a fundamental component of vascular surgery and many other fields of medicine. From central venous catheterization to complex aneurysm repair, guidewires are essential for the placement and exchange of vascular devices. As the use of implanted and endovascular devices has increased over the past several decades, so too has the potential for procedure-related complications involving retained, fractured, or embolized intravascular foreign bodies. Although retained intravascular foreign bodies are uncommon, they can present significant management challenges, particularly when the device is extensive, has migrated from its original location, or cannot be safely accessed using standard percutaneous techniques.

Chronic venous insufficiency (CVI) is the most common vascular disorder worldwide, and the most agreed-upon etiology of disease is valvular incompetence leading to pathologic venous reflux [6]. Incompetent perforator veins (IPVs) have been associated with advanced venous disease, and treatment of these pathologic perforator veins may be considered in select patients with persistent or recurrent symptomatology. There are multiple novel techniques and devices available for the treatment of incompetent perforator veins, including thermal ablation techniques that may require guidewire insertion before catheter placement [7,8].

Perforator veins are relatively small and anatomically variable vessels that traverse the deep fascia and connect the superficial and deep venous systems [9]. Their smaller caliber, variable course, and one subfascial bicuspid unidirectional valve can make guidewire manipulation technically demanding as compared with the major superficial venous trunks [9]. Because perforator veins communicate directly with the deep venous system, inadvertent advancement of equipment may allow a device introduced peripherally to migrate into larger deep veins. In the present case, the guidewire ultimately extended from the distal tibial region through the popliteal region and proximally into the femoral region, demonstrating how a device introduced during treatment of a peripheral perforator vein can become displaced extensively within the deep venous system.

Retained intravascular foreign bodies can result in complications related to thrombosis, infection, vessel injury, or migration. If a foreign body reaches the heart or pulmonary circulation, there can be potentially serious cardiopulmonary complications [10]. The clinical consequences depend on the location, size, composition, and mobility of the retained device. 

Notably, patients with retained intravascular foreign bodies may remain asymptomatic despite the presence of a potentially significant retained device [11]. In an evidence summary by Schechter et al., 135 publications comprising 574 cases of intravascular foreign bodies identified between 2000 and 2012 were reviewed, including 31 cases involving retained guidewires. Only 5.6% of patients were reported as symptomatic. Importantly, among the cases in which retrieval information was available, all 10 retained guidewires were successfully retrieved using endovascular techniques [1]. Thus, the absence of acute symptoms should not necessarily be interpreted as evidence that a retained intravascular device is clinically insignificant. This is particularly relevant to our case, in which the patient remained entirely asymptomatic despite an unusually long segment of retained guidewire extending through the deep venous system, without pain, dyspnea, or other systemic symptoms. 

Endovascular retrieval is generally preferred for intravascular foreign bodies because it avoids the morbidity associated with open exploration. Common retrieval techniques include loop snares, baskets, forceps, and balloon-assisted techniques, depending on the location and characteristics of the retained device [12]. In a review by Schechter et al., 94% of reported endovascular retrieval attempts were successful, supporting a minimally invasive approach as the preferred initial strategy when technically feasible [1]. 

In the present case, however, several factors made standard endovascular retrieval less attractive. First, the guidewire was exceptionally long, extending from the midshaft of the femur to the distal tibia. Second, extensive edema and tumescence limited the ability to visualize and characterize the wire using transcutaneous DUS. Third, adequate distal venous access through the tibial and popliteal veins could not be obtained. Finally, manipulation of a long, mobile intravascular wire through limited venous access raised concern for further migration or vessel injury. These factors collectively shifted the risk-benefit assessment away from percutaneous retrieval and toward direct surgical exploration.

Device configuration and the location of the retained wire are important when selecting a retrieval strategy. Bourke and Claydon recently described a guidewire retained at the introducer sheath during radiofrequency ablation of the great saphenous vein that was retrieved endoluminally using 5-Fr grasping forceps under ultrasound guidance [8]. Meanwhile, the guidewire in the present case had migrated extensively through the distal tibial, popliteal, and femoral venous segments, while distal venous access and control were limited. This distinction illustrates why retrieval strategies for retained guidewires should be individualized according to the location, configuration of the foreign body, and available access.

Prevention of retained intravascular foreign bodies relies on consistent procedural practices designed to maintain control of guidewires and other devices throughout the intervention. Important measures include maintaining continuous visualization and control of the guidewire, confirming wire position before advancing or withdrawing equipment, avoiding excessive advancement or withdrawal when resistance is encountered, and maintaining clear communication between the operator and assisting personnel [13]. Although the precise mechanism of guidewire loss in the present case could not be established, this complication highlights the importance of these preventive measures during technically demanding venous interventions.

The patient's postoperative course was uncomplicated, and she was discharged within 24 hours. At five-and-a-half years of follow-up, she had experienced no procedure-related complications or other untoward effects. Her persistent lower-extremity edema was attributed to her pre-existing chronic venous insufficiency rather than the guidewire retrieval. This long-term follow-up supports the effectiveness of open retrieval in this particular clinical setting and demonstrates that direct venous exploration and repair can be performed successfully when endovascular retrieval is technically unfavorable.

This case has several limitations. As a single-patient report, it cannot establish the comparative safety or superiority of open surgical retrieval over endovascular techniques. Because the initial endovenous ablation was performed at an outside practice, complete technical specifications of the guidewire and associated equipment were unavailable, limiting reconstruction of the precise mechanism of device loss. In addition, no central venous endovascular retrieval attempt was performed, and the rationale presented reflects the treating team's case-specific assessment rather than a general contraindication to percutaneous retrieval. Finally, the favorable five-and-a-half-year outcome represents an individual clinical result and should not be generalized to all retained intravascular guidewires.

## Conclusions

Retained guidewires are uncommon but potentially serious complications of endovenous procedures and should be recognized promptly. Endovascular retrieval remains the preferred approach when technically feasible because it can avoid the morbidity associated with open surgical exploration. However, the retrieval strategy should be individualized according to device length and location, available venous access, vascular anatomy, and the risk of further migration or vessel injury. In the present case, the extensive length of the retained guidewire, inability to obtain reliable distal venous access, and concern for further migration or venous injury made direct surgical retrieval the preferred approach. Open popliteal venotomy allowed successful removal of the guidewire without perioperative complications, with no procedure-related adverse effects observed at five-and-a-half-year follow-up. This case demonstrates that open surgical retrieval remains an available and important option when a percutaneous strategy is judged technically unfavorable.
